# Very High Dose Immunoglobulin Treatment for Chronic Inflammatory Demyelinating Polyneuropathy: A Multicentre UK Study

**DOI:** 10.1111/ene.70429

**Published:** 2025-11-18

**Authors:** Yusuf A. Rajabally, Joumana Freiha, Young Gi Min, Chinar Osman

**Affiliations:** ^1^ Aston Medical School Aston University Birmingham UK; ^2^ Inflammatory Neuropathy Service, Department of Neurology University Hospitals Birmingham Birmingham UK; ^3^ Wessex Neurological Centre University Hospital Southampton Southampton UK; ^4^ Department of Neurology Seoul National University College of Medicine Seoul Republic of Korea; ^5^ Department of Neurology Severance Hospital, Yonsei University College of Medicine Seoul Republic of Korea

**Keywords:** chronic inflammatory demyelinating polyneuropathy, immunoglobulin, outcome, severity, very high dose

## Abstract

**Background:**

Immunoglobulin dosing is individualised in chronic inflammatory demyelinating polyneuropathy (CIDP).

**Methods:**

We retrospectively compared differences in presentation/outcomes/side effects in subjects on very high dose immunoglobulin defined as ≥ 2 g/kg every 3 weeks (‘Group A’) and subjects on ≤ 1 g/kg every 3 weeks (‘Group B’), from 2 UK centres.

**Results:**

One‐hundred and eight subjects with CIDP received immunoglobulins. Group A consisted of 12 subjects (11.1%). Mean dose was 2.63 g/kg every 3 weeks (SD: 0.71). Six subjects (50%) had typical CIDP, 3 (25%) had motor CIDP, and 3 (25%) had multifocal CIDP. Group B consisted of 40 subjects (37%) on a mean dose of 0.47 g/kg every 3 weeks (SD: 0.16). Compared to subjects from Group B, subjects from Group A had greater pre‐treatment disability (*p* = 0.029), more common associated autoimmune disease (*p* = 0.034), worse post‐treatment outcome (*p* = 0.005) and a longer time to maximal improvement (*p* = 0.041). No differences were found between the two groups for age/gender/weight/acuteness of presentation/side‐effects. Occurrence of any side‐effect (*p* = 0.005), and of thromboembolic complication (*p* = 0.022), were associated with presence of another autoimmune disease.

**Conclusions:**

Very high dose immunoglobulin may be partially effective in a minority of subjects with CIDP. Subjects treated with very high dose immunoglobulin may have greater pre‐treatment disability, be more likely to have another autoimmune disease, have worse post‐treatment outcomes, and take longer to reach maximal improvement, than subjects on lower doses. Concurrent autoimmune disease may increase immunoglobulin‐induced thromboembolic risk. Earlier consideration of alternative therapies may be more appropriate than immunoglobulin dose escalation in subjects with suboptimal immunoglobulin response.

## Graphical abstract

Immunoglobulin dosing is individualised in chronic inflammatory demyelinating polyneuropathy (CIDP). Comparing subjects on very high dose immunoglobulin, defined as ≥ 2 g/kg every 3 weeks, and subjects on ≤ 1 g/kg every 3 weeks from 2 UK centres, we found the former had greater pre‐treatment disability, more common associated autoimmune disease, worse post‐treatment outcomes and took longer to improve. Other treatments may be more appropriate than very high dose immunoglobulin in case of insufficient immunoglobulin response.

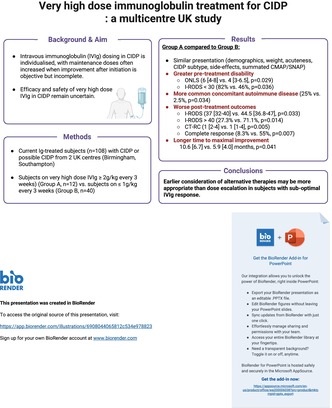

## 1 Introduction

The treatment of chronic inflammatory demyelinating polyneuropathy (CIDP) with intravenous immunoglobulin (IVIg) or subcutaneous immunoglobulin (SCIg) is supported by an evidence base of multiple randomised controlled trials (RCTs) [1]. The initiating dose is widely accepted as 2 g/kg, administered over 5 days, which comes from previous therapeutic trials in CIDP, although originating from the dose administered in idiopathic thrombocytopenic purpura in children, first described over 40 years ago [2]. A commonly used long‐term maintenance post‐initiation dose is 1 g/kg every 3 weeks, based on the ‘Intravenous immune globulin (10% caprylate‐chromatography purified) for the treatment of chronic inflammatory demyelinating polyradiculoneuropathy’ (‘ICE’) study [3]. Other therapeutic protocols have also been proposed both for initiation and maintenance [4, 5]. While individualised dosing for IVIg is recommended and supported by guidelines, practices worldwide show considerable variation [6]. Few studies have shown the individualised nature of immunoglobulin dose and frequency requirements in subjects with CIDP [7]. Doses required for long‐term maintenance have otherwise been found not to correlate with actual body weight or disease severity at onset [7, 8, 9]. In the UK, the dose of treatment initiation is now calculated through ideal body weight rather than actual weight, and regular attempts at dose reduction are recommended by the Department of Health Commissioning Guidelines [10], with the aim of finding the lowest effective dose in each subject.

Through the application of our IVIg treatment protocol, we previously found in a cohort of 47 subjects at our unit in Birmingham, that the average long‐term dosing requirements were about two‐thirds that of the maintenance dose of the ICE Study [5]. However, we also observed wide variations in dosage with the highest dose used reaching 3.84 g/kg every 3 weeks. Similar observations were made in Southampton with the application of the ICE Study protocol for maintenance. In both our units, in the presence of objective but incomplete, outcome‐measured improvement post‐IVIg initiation, the IVIg dose is increased, before consideration of other treatment options. As such, in Birmingham an initial partial, clinically meaningful, IVIg response of insufficient amplitude at a dose of 2 g/kg administered for the first two courses [5], leads to dose escalation, accompanied by an increase in frequency in case of early wearing off. In Southampton, improvement of any amplitude after initiation with two courses of immunoglobulin at 2 g/kg leads to an initial dose reduction to 1 g/kg every 3 weeks, as per the ICE Study protocol, following which, in case of decline, the dose is re‐increased up to the level required to, (i) reach the previously attained level after initiation, and then subsequently (ii) maximise the achievable benefit. In both our units, serial assessments through outcome measures are systematically performed between 2 and 4 weeks (i) after the 2 initial immunoglobulin infusions and subsequently, (ii) after each dose alteration.

The Progress in CIDP (ProCID) trial investigated through a prospective, randomised, parallel‐group multicentre study, IVIg doses of 0.5, 1 and 2 g/kg every 3 weeks [11]. IVIg responder rates were of 65%, 80% and 92% respectively in the three groups, with differences however reaching significance between the lowest and the highest dose only. The ProCID trial demonstrated that a proportion of subjects with CIDP require very high doses of IVIg for a minimal response. This appears in keeping with our experience, that in a proportion of subjects, the IVIg response may be incomplete at initiation, with further amelioration occurring subsequently from dose escalation. Whether responders to very high doses are different from responders to standard doses of immunoglobulin in terms of demographics and disease characteristics, treatment effectiveness and side effects is unknown. IVIg is a high‐cost therapy with potentially serious, in particular thromboembolic complications [12], and better understanding of the eventual specificities of the minority of subjects appearing to benefit from very high dose immunoglobulin, is desirable.

We aimed to study a cohort of subjects from our centres who required mean maintenance doses of immunoglobulin ≥ 2 g/kg every 3 weeks through the application of our clinical protocols, and to compare their demographic and disease characteristics with those of consecutive subjects treated at our centres, requiring a mean of ≤ 1 g/kg every 3 weeks for maintenance.

## 2 Materials and Methods

We retrospectively reviewed records of all current immunoglobulin‐treated subjects with a diagnosis of CIDP or possible CIDP, meeting European Academy of Neurology/Peripheral Nerve Society (EAN/PNS) 2021 criteria [13], attending University Hospitals Birmingham and University Hospital Southampton, UK. Inclusion required being currently on IVIg or SCIg, not having any concurrent other immunomodulatory or immunosuppressive therapy for CIDP, and having been clinically stable through objective outcome‐measured evaluation, for at least 3 months.

We collected demographics, disease sub‐type, mode of onset, pre‐treatment disease duration from symptom onset, pre‐treatment Overall Neuropathy Limitation Score (ONLS), and the best‐achieved ONLS on maintenance treatment, for subjects on a mean maintenance immunoglobulin dose ≥ 2 g/kg every 3 weeks. This dosing group was defined as ‘Group A’. The mean 3‐weekly dose was calculated from the actual frequency of administration in each subject, which had been individualised according to the timing of wearing off of treatment effects. The ONLS is systematically utilised at clinic attendances in our practices (five points for upper limb score, seven points for lower limb score; optimal score of 0). We ascertained post‐treatment outcome through the ‘CIDP Treatment‐Response Category’ (‘CT‐RC’), which is constructed on an exclusively result‐based categorical classification of response, considering treatment‐induced ONLS sub‐score changes achieved in the upper and lower limbs [14]. We also collected concurrent I‐RODS scores [15], pre‐ and post‐treatment. We in addition, aimed to determine secondarily, associations of pre‐treatment electrophysiological measures with immunoglobulin dose requirements. Pre‐treatment summated compound muscle action potential values (ƩCMAP) were recorded, adding the distal CMAP evoked for unilateral median/ulnar/common peroneal/tibial nerves as were summated sensory nerve action potential values (ƩSNAP), adding unilateral sural and radial SNAPs.

We collected similar data for subjects with CIDP treated at our centres, on a mean dose of immunoglobulin ≤ 1 g/kg every 3 weeks, similarly calculated through the actual frequency of infusion in each subject. This dosing group was defined as ‘Group B’. We excluded from the current analysis subjects on mean doses of 1 to 2 g/kg every 3 weeks. This was decided in an attempt to better ascertain differences between the two studied dose categories, in analogy with the findings of the ProCID study which found no differences in responder rates comparing 1 vs. 2 g/kg groups, nor of those comparing 0.5 to 1 g/kg groups. We performed comparative studies of the two groups for relevant co‐variables including age, gender, weight, disease subtype, acuteness of presentation, pre‐ and post‐treatment disability, associated autoimmune diseases, associated diabetes, associated concurrent or previous malignancy, any side effect, as well as, specifically, thromboembolic complications. Association studies were performed to determine the correlates of any side effect and of thromboembolic complication in the combined cohort.

Statistical analyses were performed with R version 4.3.2 and SPSS 28.0 (Armonk, USA). Continuous variables were presented in median (interquartile range), while categorical variables were in number (%). To compare means or frequencies between groups Mann–Whitney U test or Fisher's exact test was used, respectively. Change in ONLS or I‐RODS following treatment was analysed using the paired Wilcoxon test, where multiple comparisons were adjusted with the Benjamini‐Hochberg method. Correlations were studied through Spearman's Rank Correlations. Corrections for multiple correlations were not performed in view of the exploratory nature of this analysis. Independent associations were sought through logistic regression, including in models the co‐variables having demonstrated significant correlations. Significance was set at *p* < 0.05 for all tests.

This analysis was conducted as part of registered and approved retrospective clinical audits of the diagnosis and management of CIDP at University Hospitals Birmingham (CARMS‐20702) and University Hospital Southampton (SEV‐8371), UK. Audit does not require Ethics Committee approval in the UK.

## 3 Results

### 3.1 Study Subjects

One‐hundred and eight subjects were on current immunoglobulin treatment for CIDP at our two centres at the time of data collection. All met EAN/PNS 2021 criteria for CIDP or possible CIDP. Group A consisted of 12 subjects (11.1%) and Group B of 40 subjects (37%). Fifty‐six subjects (51.9%), on a dose of 1–2 g/kg every 3 weeks, were excluded from further analysis.

### 3.2 Baseline Characteristics

Baseline clinical characteristics of the two groups are summarised in Table 1. Median age was comparable between Group A and Group B (52 [40–71] vs. 60 [54–68], *p* = 0.4). The gender distribution was also similar (female 50% vs. 35%, *p* = 0.5). There were no significant differences in disease duration before treatment (*p* = 0.5), clinical subtype (typical: *p* = 0.9; motor: *p* = 0.074), acuteness of onset (*p* = 0.6), or comorbidities such as diabetes (*p* > 0.9), or malignancy (*p* = 0.3). Summated CMAP and SNAP values were also equivalent between groups (*p* = 0.6 and *p* = 0.6, respectively). In contrast, pre‐treatment ONLS scores were significantly higher in Group A (6 [4–8] vs. 4 [3–6.5], *p* = 0.029). All subjects in Group A had pre‐treatment ONLS of ≥ 4. Pre‐treatment I‐RODS was also lower in Group A (21 [13–27] vs. 31 [17–39]), although this did not reach statistical significance (*p* = 0.2). However, the proportion of patients with severe disability (I‐RODS < 30) was higher in Group A (9/12, 82% vs. 17/40, 46%; *p* = 0.036). In addition, concomitant autoimmune diseases were more frequent in Group A (3/12, 25% vs. 1/40, 2.5%; *p* = 0.034). Specifically, the three subjects from Group A with another concurrent autoimmune disease had systemic lupus erythematosus (SLE), mixed connective tissue disease (MCTD), and rheumatoid arthritis (RA), whereas the subject from Group B had Hashimoto's thyroiditis.

**TABLE 1:** Comparison of pre‐ treatment characteristics between subjects who required an immunoglobulin dose ≥ 2 g/kg every 3 weeks (Group A) and those who received an immunoglobulin dose of ≤ 1 g/kg every 3 weeks (Group B) at University Hospitals Birmingham and University Hospital Southampton, UK.

	Group A (≥ 2 g/kg every 3 weeks) (*n* = 12)	Group B (≤ 1 g/kg every 3 weeks) (*n* = 40)	*p*
Age—years (range)	52 (40–71)	60 (54–68)	0.4
Female—*n* (%)	6 (50%)	14 (35%)	0.5
Disease duration pre‐treatment (months)	12 (5–39)	20 (6–84)	0.5
Subtype—*n* (%)			
Typical	6 (50%)	19 (48%)	0.9
Motor	3 (25%)	2 (5%)	0.074
Acuteness of onset—*n* (%)	2 (17%)	4 (10%)	0.6
Mean maintenance dose of immunoglobulin—g/kg every 3 weeks (SD)	2.63 (0.71)	0.47 (0.16)	**< 0.001**
Pretreatment ONLS	6 (4–8)	4 (3–6.5)	**0.029**
Pretreatment I‐RODS	21 (13–27)	31 (17–39)	0.2
Pre‐treatment I‐RODS < 30	9 (82%)	17 (46%)	**0.036**
Comorbidity—*n* (%)			
Diabetes	0 (0%)	1 (2.5%)	> 0.9
Malignancy	1 (8.3%)	3 (7.5%)	0.3
Other autoimmune diseases	3 (25%)	1 (2.5%)	**0.034**
Summated CMAP (mV)	20 (8–32)	26 (11–33)	0.6
Summated SNAP (μV)	23 (11–36)	17 (2–37)	0.6

*Note:* Bold values denote statistical significance.

### 3.3 Efficacy of Escalation to Very High Dose Immunoglobulin

**FIGURE 1:**
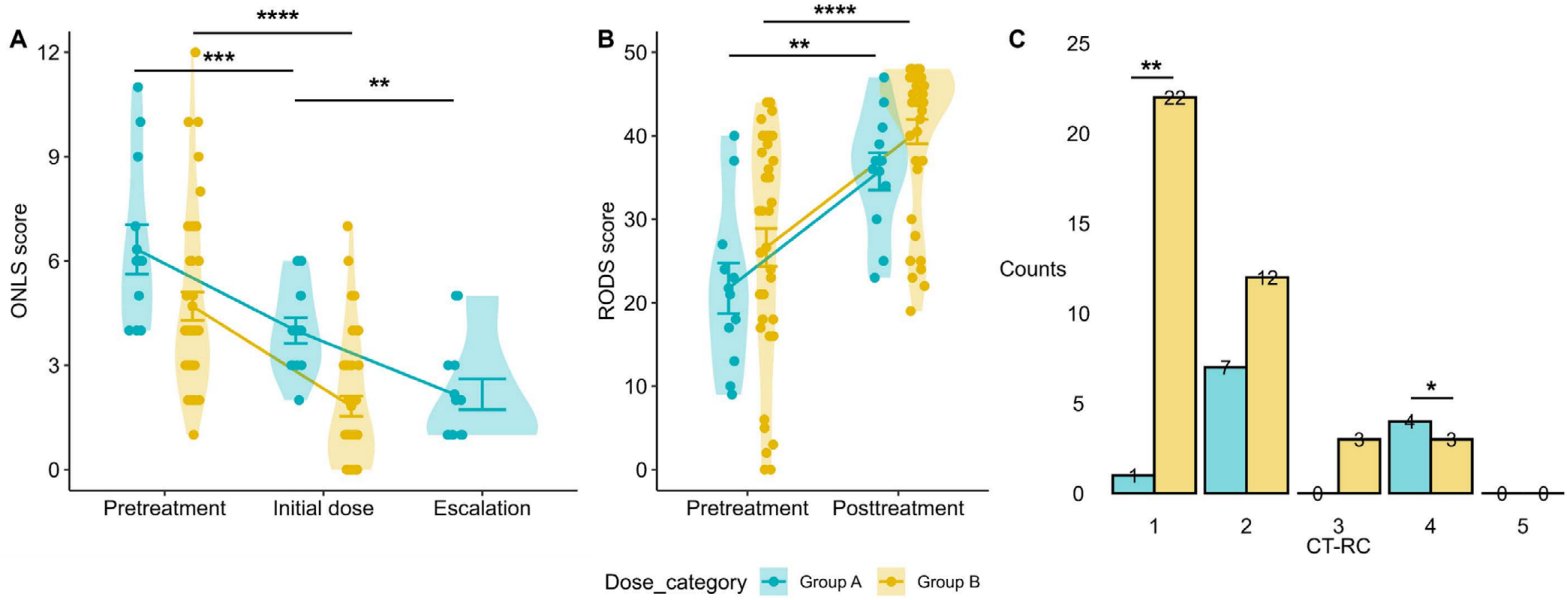
Outcomes after initial treatment and after dose escalation in 12 subjects with CIDP on ≥ 2 g/kg every 3 weeks (Group A) vs. outcomes with usual treatment protocol in 40 subjects on ≤ 1 g/kg every 3 weeks (Group B), at University Hospitals Birmingham and University Hospital Southampton, UK: (A) ONLS score changes in Group A and Group B from pre‐treatment level with initial treatment (both groups) and after immunoglobulin dose escalation (in Group A only). (B) I‐RODS raw score changes (pre‐treatment and after initiation in Group B, pre‐treatment after immunoglobulin dose escalation in Group A). (C) CT‐RC category comparison for Group A (after immunoglobulin dose escalation) vs. Group B (after initial treatment). (*p < 0.05; **p < 0.01; ***p < 0.001; ****p < 0.0001).

Findings are summarised in Table 2. Pre‐treatment ONLS scores, during initial dose immunoglobulin induction/maintenance, and after dose escalation (for Group A) are shown in Figure 1A. Immunoglobulin initiation resulted in significant improvements compared with baseline in both groups (Group A: 6 [4–8] → 4 [3–5], *p* < 0.001; Group B: 4 [3–6.5] → 1 [0–3], *p* < 0.0001). However, Group A still exhibited substantially greater disability after initiation (4 [3–5] vs. 1 [0–3], *p* < 0.001). Dose escalation significantly improved disability in Group A (4 [3–5] → 1.5 [1–3], *p* < 0.01). Following dose escalation, all patients in Group A showed at least a one‐point improvement in ONLS, with 50% of patients achieving an improvement of two points or more.

**TABLE 2:** Comparison of post‐treatment outcomes between subjects who required an immunoglobulin dose ≥ 2 g/kg every 3 weeks (Group A) and those who received an immunoglobulin dose of ≤ 1 g/kg every 3 weeks (Group B) at University Hospitals Birmingham and University Hospital Southampton, UK.

	Group A (≥ 2 g/kg every 3 weeks) (*n* = 12)	Group B (≤ 1 g/kg every 3 weeks) (*n* = 40)	*p*
ONLS achieved after initial immunoglobulin treatment	4 (3–5)	1 (0–3)	**< 0.001**
Final I‐RODS	37.0 (32.0–40.0)	44.5 (36.75–47)	**0.033**
Proportion of subjects with final raw I‐RODS > 40	3/11 (27.3%)	27/38 (71.1%)	**0.014**
Final CT‐RC	1 (2–4)	1 (1–4)	**0.005**
Proportion of final CT‐RC of			
1 (Complete response)	1/12 (8.3%)	22/40 (55%)	**0.007**
2 (Good partial response)	7/12 (58%)	12/40 (30%)	0.095
3 (Moderate partial response)	0/12 (0%)	3/40 (7.5%)	1.000
4 (Poor partial response)	4/12 (33%)	3/40 (7.5%)	**0.041**
5 (Non‐responsive)	0/12 (0%)	0/12 (0%)	1.000
Mean time to maximal improvement—months (SD)	10.58 (6.87)	5.86 (3.99)	**0.041**

*Note:* Bold values denote statistical significance.

Immunoglobulin treatment resulted in significant improvements in final I‐RODS scores in both groups (Group A: 21 [13–27] → 37 [30–41], *p* < 0.01; Group B: 31 [17–39] → 45 [37–47], *p* < 0.0001) (Figure 1B). The magnitude of improvement was comparable (median change: 11 [4–28] vs. 9 [5–20], *p* = 0.8), and the proportion of clinically meaningful improvement, as defined by the published minimal clinically important difference (MCID) cut‐off of ≥ 4 raw points [16] was also similar (10/12, 91% vs. 32/40, 86%, *p* > 0.9). Subjects in Group A however had a lower final post‐treatment I‐RODS compared to those in Group B (37 [32–40] vs. 44.5 [36.75–47]; *p* = 0.033). The proportion of subjects with post‐treatment I‐RODS > 40 was also lower in Group A compared to Group B (3/11, 27.3% vs. 27/38, 71.1%; *p* = 0.014).

Distribution of CT‐RC according to treatment is shown in Figure 1C. Final outcome ascertained through the CT‐RC was worse in subjects in Group A compared to Group B (1 [2‐4] vs. 1 [1‐4]; Mann Whitney *U* Test: *p* = 0.005). The proportion of CT‐RC of 1, representing a complete response, was higher in Group B (22/40, 55% vs. 1/12, 8.3%, *p* = 0.007), whereas the proportion of poor partial response (CT‐RC of 4) was higher in Group A (4/12, 33.3% vs. 3/40, 7.5%, *p* = 0.042).

The time to maximal improvement, from first immunoglobulin infusion, was longer in Group A compared to Group B (10.58 months [SD: 6.67] vs. 5.86 months [SD: 3.99]; *p* = 0.041).

### 3.4 Safety of Escalation to Very High Dose Immunoglobulin

Inter‐group comparative results are shown in Table 3. The complication rate did not differ between the two groups (any side effect: 2 [17%] vs. 9 [23%], *p* > 0.9; thromboembolic event: 1 [8.3%] vs. 1 [2.5%], *p* = 0.4). Occurrence of any side effect to immunoglobulin therapy correlated with the motor CIDP phenotype (*p* = 0.025) and presence of another concurrent autoimmune disease (*p* = 0.005). An independent association of any side effect was found only with the presence of another concurrent autoimmune disease (*p* = 0.005). Occurrence of thromboembolic complication attributable to immunoglobulin treatment correlated only with the presence of another concurrent autoimmune disease (*p* = 0.022).

**TABLE 3:** Comparison of side‐effects and thromboembolic complications to immunoglobulin in subjects who required ≥ 2 g/kg every 3 weeks (Group A) and those who received an immunoglobulin dose of ≤ 1 g/kg every 3 weeks (Group B) at University Hospitals Birmingham and University Hospital Southampton, UK.

Side effect	Combined cohort (*n* = 52)	Group A (*n* = 12)	Group B (*n* = 40)	*p*
Any side effect	11 (21%) (Headache, skin rash)	2 (17%)	9 (23%)	> 0.9
Thromboembolism	2 (3.8%) (Pulmonary Embolism, Deep Vein Thrombosis)	1 (8.3%)	1 (2.5%)	0.4

## 4 Discussion

Individualised immunoglobulin dosing is routinely practised for CIDP with wide dose ranges. There is a paucity of data on the use of very high doses of immunoglobulin in routine care. To our knowledge, only two case series from France and the UK have described the use and benefit of very high dose immunoglobulin [17, 18]. The doses used in these case series were heterogeneous. Whereas the French study reported a mean dose of 2.63 g/kg every 3 weeks in four subjects [18], the UK study described four subjects with CIDP on doses of 3.75, 6.75, 1.50 and 5.20 g/kg every 3 weeks, respectively, at their last documented review [17]. A recent immunoglobulin dose‐comparative study, the ProCID trial [11], has otherwise shown that a proportion of subjects with CIDP require up to 2 g/kg every 3 weeks for an objective response, implying a higher global response rate at that dose compared to 0.5 g/kg every 3 weeks. This is consistent with the clinical observations of continuing amelioration with escalation to very high dose in some subjects with CIDP.

In the current study, we defined ‘very high dose’ immunoglobulin as ≥ 2 g/kg every 3 weeks, based on the ProCID study protocol. The dose used in each subject from both studied groups, was importantly, attained progressively, through treatment individualisation, itself based on outcome‐measured response. Dose dependency was then confirmed through attempted dose reduction in every patient. The mean dose used in this group (Group A) was coincidentally perfectly identical to that described in the French case series (2.63 g/kg every 3 weeks) and the maximal dose used was just under 4 g/kg every 3 weeks. Excluding from the previous UK series the subject who was on 1.50 g/kg every 3 weeks [17], and who therefore did not meet our definition of very high dose, the 3 remaining subjects of that series received a mean 3‐weekly of 5.23 g/kg every 3 weeks.

Through comparison with subjects on ≤ 1 g/kg every 3 weeks, with the objective of detecting relevant differences in analogy with findings of ProCID, we found that subjects treated with very high dose immunoglobulin had greater pre‐treatment disability. The previous two case series described comparable pre‐treatment disability levels to ours, with an ONLS ≥ 6 in 6/8 subjects, and MRC sum scores of 49/70 and 39/70 in the remaining 2 [17, 18]. This may intuitively be explained by a greater need for clinical amelioration resulting in dose escalation supported by initial partial benefit. Subjects in Group A also had another concurrent autoimmune disease more commonly than those in Group B. Concurrent autoimmune disease was reported in 1/4 subjects in the UK study [17], and in 2/4 subjects in the French series [18]. Hence, the two previous reports describe in combination, comparable proportions of associated autoimmune disorders, to that of our cohort of subjects on very high dose immunoglobulin. These findings may suggest different pathophysiological disease mechanisms in CIDP associated with other autoimmune conditions, resulting in poorer response to usual lower immunoglobulin dosing regimens. Disappointingly, we found that subjects from Group A had poorer final outcomes after escalation, compared to subjects from Group B, who did not require escalation. This may similarly suggest pathophysiological specificities in a minority of subjects with CIDP, with poor partial immunoglobulin response, only incompletely overcome despite dose escalation. As shown by the contrasting recent studies of rituximab in CIDP, found helpful in immunoglobulin refractory [19] but not in immunoglobulin responsive subjects [20], variable underlying mechanistic processes may be at play in CIDP and as a result, explain heterogeneous treatment responses. Of note, subjects from Group A achieved similar post‐treatment outcomes to those of the French series [18], (mean post‐treatment ONLS: 2.17 vs. 2.75). Comparison with the UK series was not possible due to the variable outcome measures used in that study, although only one of the three subjects having received ≥ 2 g/kg every 3 weeks in that series appeared to have been left with significant weakness and disability from the descriptions provided [17].

An important difference between our study and the previously reported cases relates to the associated treatments attempted. Our patients on very high dose were importantly all on their first CIDP treatment with immunoglobulin monotherapy whereas those of the French study all had prior trials of variable combinations of corticosteroids, plasma exchange, azathioprine, rituximab, mycophenolate mofetil and cyclophosphamide [18]. Only one of the four subjects from the UK series had received only immunoglobulin, one having received plasma exchange, one mycophenolate mofetil and one multiple treatments including corticosteroids, plasma exchange, methotrexate, cyclosporin, autologous stem cell transplant and alemtuzumab [17]. In contrast, the findings in our cohort provide an indication of the value of escalation to very high dose immunoglobulin monotherapy in earlier disease, unaffected by additional treatments that may obscure the interpretation of presumed respective treatment effects. In this regard, it is noteworthy that the globally very positive outcomes suggested in the UK case series cannot be attributed with certainty to high dose immunoglobulin usage, given the multiple concurrent treatments administered in these subjects.

We also found that time to maximal improvement was longer in Group A (> 10 months) compared to Group B (< 6 months). Although consistent with our observations of continuing improvement with dose escalation in Group A, this also demonstrates another important limitation of very high dose immunoglobulin, which only provides its maximal effects with considerable delay. It is however possible that this delay may be mainly due to the gradual dose escalation performed in subjects with suboptimal response, and that quicker maximal effects may have been obtained through more rapid escalation to higher dosage.

The association of immunoglobulin‐induced thromboembolic complications with the presence of another concurrent autoimmune disease is in keeping with existing literature indicating a higher thrombotic risk in SLE [21], RA [22] and Sjögren's syndrome [23]. As subjects in Group A had another concurrent autoimmune disorder more commonly than those in Group B, this association is concerning. Even though previous studies have not linked the total dose of immunoglobulin to an increased risk of thrombosis [24, 25], considering other treatment options early on might be safer and more appropriate. Of note, the comparable rate of any side effect in Group A and Group B is in keeping with the findings of ProCID, which also found no inter‐group difference except for headaches [11]. It is otherwise possible that the absence of an inter‐group difference in headache occurrence may be explained in our cohort by the advice provided in our practices, about prophylactic use of analgesics pre‐infusion, from early treatment stages, which may have resulted in better tolerance and/or under‐reporting of headaches in subjects on very high dose immunoglobulin.

Our study has several limitations including its retrospective design and small numbers of included subjects, related both to the low prevalence of CIDP and the rarity of very high dose immunoglobulin usage. We arbitrarily decided to define dosing groups based on the design of the ProCID study and in analogy with its results, which may have affected our findings. Our study did not include ultra‐high immunoglobulin doses > 4 g/kg every 3 weeks as described in the previous UK series, as such dosing is not used at our centres. The benefit, cost/benefit ratio and safety profile of ultra‐high dose regimens however raise the same questions, although probably with more serious concerns, as with very high dose treatment. Finally, the potential roles in our findings of anchoring bias from the treating neurologist's perspective [26], and of non‐compliance to proposed treatments from that of the patient [27], were not considered.

We believe that our study, despite its limitations, yields important novel data regarding the use of very high dose immunoglobulin, as initial monotherapy for CIDP. Earlier reports had, despite multiple caveats including the administration of concurrent therapies, supported its use, highlighting its benefits and favourable safety profile. However, we found that while it may offer therapeutic value to a subset of patients with significant baseline disability, subsequently best achieved outcomes were inferior and attained with greater delay, compared to those with doses ≤ 1 g/kg every 3 weeks. Furthermore, while thromboembolic events were not associated with very high dose immunoglobulin use, they were associated with the presence of another concurrent autoimmune disease, a situation where very high dose immunoglobulin may commonly be required. In conclusion, our findings raise new questions on the appropriateness of very high dose immunoglobulin as a therapeutic option for CIDP and may favour earlier consideration of potentially more effective as well as safer treatment alternatives.

## Author Contributions

**Yusuf A. Rajabally:** conceptualization; writing original draft; methodology; formal analysis; project administration; supervision. **Joumana Freiha:** formal analysis; methodology; project administration; writing of original draft; **Young Gi Min:** formal analysis; methodology. **Chinar Osman:** formal analysis; writing – review and editing.

## Conflicts of Interest

Y.A.R. has received consultancy honoraria from Sanofi, Argenx, Janssen, LFB, Polyneuron, Grifols, Takeda, Dianthus, Vitaccess, has received educational sponsorships from LFB and CSL Behring and has obtained research grants from LFB. C.O. has received speaker/consultancy honoraria from Takeda, Grifols and Terumo BCT. J.F. and Y.G.M. have no disclosures.

## Data Availability

The data that support the findings of this study are available on request from the corresponding author. The data are not publicly available due to privacy or ethical restrictions.
